# Nurse-Led, Shared Medical Appointments for Common Gastrointestinal Conditions—Improving Outcomes Through Collaboration With Primary Care in the Medical Home: A Prospective Observational Study

**DOI:** 10.1093/jcag/gwy061

**Published:** 2018-10-24

**Authors:** Kerri L Novak, Jennifer Halasz, Christopher Andrews, Colleen Johnston, Willem Schoombee, Divine Tanyingoh, Gilaad G Kaplan, Sander Veldhuyzen van Zanten, Mark Swain

**Affiliations:** 1 University of Calgary, Alberta, Canada; 2 Division of Gastroenterology and Hepatology, University of Calgary, Calgary, Alberta, Canada; 3 Division of Gastroenterology and Hepatology, Department of Medicine, University of Calgary, Calgary, Alberta, Canada; 4 Alberta Health Services, Calgary, Alberta, Canada; 5 Foothills Primary Care Network, Calgary, Alberta, Canada; 6 Division of Gastroenterology and Hepatology and Department of Community Health Sciences, University of Calgary, Calgary, Alberta, Canada; 8 Division of Gastroenterology, University of Alberta, Edmonton, Alberta, Canada

**Keywords:** Dyspepsia, Gastroesopheageal reflux, Medical home, Shared medical appointment

## Abstract

**Background:**

Gastroesophageal reflux disease (GERD), dyspepsia and irritable bowel syndrome (IBS) are common gastrointestinal disorders accounting for a significant demand for specialty care. The aim of this study was to evaluate safety, access and outcomes of patients assessed by a nurse-led, shared medical appointment.

**Methods:**

This prospective observational study utilized a sample of 770 patients referred to a gastroenterology Central Access and Triage for routine GERD, dyspepsia or IBS from 2011 to 2014. Patient demographics, clinical indication, frequency and outcomes of endoscopy, quality of life, wait times and long-term outcomes (>2 years) were compared between 411 patients assigned to a nurse-led, shared medical appointment and 359 patients assigned to clinic for a gastroenterology physician consultation.

**Results:**

The nurse-led, shared medical appointment pathway compared with usual care pathway had shorter median wait times (12.6 weeks versus 137.1 weeks, P < 0.0001), fewer endoscopic exams (50.9% versus 76.3%, P < 0.0001), less gastroenterology re-referrals (4.6% versus 15.6%, P < 0.0001), and reduced visits to the emergency department (6.1% versus 12.0%, P = 0.004). After two years of follow-up, outcomes were no different between the pathways.

**Conclusions:**

Patients with GERD, IBS or dyspepsia who attend the nurse-led, shared medical appointment have improved access to care and reduced resource utilization without increased risk of significant gastrointestinal outcomes after two years of follow-up.

Gastroesophageal reflux disease (GERD), dyspepsia and irritable bowel syndrome (IBS) are among the most common referrals to gastroenterology (GI) (1–3). Wait times to see a gastroenterologist in Canada are long and increasing over time as demand continues to exceed capacity (4).

The diagnosis of IBS and dyspepsia has shifted from one of exclusion to a positive diagnostic strategy based on stereotypical clinical symptoms (5). Similarly, guidelines for GERD support evaluation of characteristic symptoms followed by a medical trial of acid suppression (6–8). Existing guidelines do not support endoscopy for the investigation of patients with IBS, uncomplicated GERD responsive to proton-pump inhibition (PPI), nor dyspepsia in the absence of alarm symptoms (anemia, recurrent vomiting, dysphagia, jaundice or weight loss) (7, 9, 10). In fact, when endoscopy is performed to investigate these common conditions, there are no clinically important changes in symptoms, patients’ overall prognosis or future management (10, 11).

Shared medical appointments present an enticing model for innovative delivery of health care, with a focus on patient education, empowerment and engagement. Shared medical appointments have been successfully implemented for smoking cessation, chronic pain and diabetes (12). Community-based, multidisciplinary sessions led by nurses improve patient knowledge, symptoms, and self-management techniques (13–15). Integration of primary and specialty care within the primary care medical home for chronic disease management is important, with increased access and patient satisfaction (16). The aim of this study was to determine if a nurse-led, shared medical appointment was a safe, accessible and feasible approach to evaluate and manage GI patients.

## METHODS

### Setting and Design

We conducted a prospective observational study in the Calgary Zone, an area with a population of 1.23 million. The Division of Gastroenterology and Hepatology (GI) at the University of Calgary accepts referrals through a single point of entry referral model: Central Access and Triage. In the Calgary Zone, 33 out of 45 practices (73% of gastroenterologists) participate in Central Access and Triage. The remaining 12 gastroenterologists (27%) are in private practice and receive referrals independently of Central Access and Triage. Wait times within private practice in the Calgary Zone are unknown. Central Access and Triage receives approximately 1700 referrals per month, with median wait times exceeding 24 months for non-urgent referrals. Primary care in the Calgary Zone is organized into five urban geographic regions and two rural regions, all referred to as Primary Care Networks.

### Study Population

Referrals to Central Access and Triage for GERD, dyspepsia or IBS were identified from November 1, 2011, to October 31, 2014, by the triage registered nurse (CJ). The selection of appropriate referrals for the program occurred within GI Central Access and Triage and not through primary care. One Primary Care Network (Calgary Foothills) was chosen as the intervention population for this study, and all patients with residential postal codes for that area were assigned to an interventional nurse-led, shared medical appointment, whereas patients from other Primary Care Networks were directed to usual care. The Foothills Primary Care Network was chosen because of the presence of existing, multidisciplinary shared medical appointments in tobacco cessation and chronic pain, with interest and willingness to engage in gastroenterology, given challenges with long wait times.

A registered nurse from GI Central Access and Triage reviewed all referrals and conducted telephone interviews to confirm eligibility. The inclusion criteria for either the nurse-led, shared medical appointment pathway or usual care pathway included (1) referral by primary care specifically for GERD, IBS, dyspepsia, abdominal pain or epigastric pain and (2) adult patients >18 years of age. Referrals with any of the following criteria were excluded from enrollment into a care pathway: (1) abnormal laboratory values (e.g., anemia, iron deficiency, elevated C-reactive protein or positive celiac screen); (2) reports of alarm features as identified by phone (e.g., weight loss, rectal bleeding, night time symptoms); (3) abnormal gastrointestinal imaging (e.g., computed tomography [CT], ultrasound); (4) coexisting gastrointestinal disorder (e.g., celiac disease, inflammatory bowel disease, family history of significant gastrointestinal disease); and (5) unable or unwillingness to participate in a shared medical appointment (e.g., mental illness, non-English speaking). Referrals with alarm symptoms were triaged for urgent evaluation by a gastroenterologist (Appendix 1).

### Intervention

The GI-experienced registered nurse responsible for leading the shared medical appointments contacted all patients who met criteria for inclusion to confirm eligibility. A detailed phone-history was taken to solicit clinical symptoms, comorbidities, medication use and prior investigations. In addition, participants completed the validated global overall symptom score for dyspepsia and quality of life survey over the phone (Short Form or SF-12) before the nurse-led, shared medical appointment (Appendix 2 and 3) (17). At the appointment within the Primary Care Medical Home, patients signed both a confidentiality agreement and consent. The nurse facilitated the education session within a Calgary Foothills Primary Care Network clinic meeting room (Appendix 4). The multidisciplinary team included a pharmacist, a behavioral change consultant to support behavior change, and a dietitian providing nutritional expertise—all employed of the Primary Care Network. Upon completion of the group session, each patient was individually assessed by either a Calgary Foothills primary care physician with interest in gastroenterology (WS) or a gastroenterologist (KN, CA). Six months after their appointment, patients were followed up by telephone by the nurse, where they voluntarily completed a repeat of the global overall symptom score and SF-12 surveys.

### Usual Care

Patients outside of the Calgary Foothills Primary Care Network entered Central Access and Triage through the traditional triage pathway. In the usual care arm, investigations and management occur at the discretion of the attending gastroenterologist or usual care. Wait times in this pathway reflect current demands and priorities allocated for referrals. Usual care pathway patients received the global overall symptom score and SF-12 survey by mail at the time of referral. After six months, repeat surveys were mailed to all patients who previously completed the baseline assessment. Information on wait times for consultation, endoscopy if performed and diagnostic outcome were also collected. In addition, all patients referred for the same clinical indication to a physician in or outside of Central Access and Triage and visits to the Emergency Department (through Alberta Health Services Analytics, Data Integration, Measurement and Reporting) for the same clinical indication in the Calgary Zone were collected.

Chart review was completed for all patients in the intervention arm from time of referral to December 31, 2016, to obtain endoscopy and relevant diagnostic imaging reports, emergency department visits and re-referrals to gastroenterology. Charts from patients from usual care were not readily accessible. Significant outcomes were defined as those significantly altering diagnosis or management (Appendix 5).

### Outcomes

The primary outcomes include wait times for consultation, endoscopic occurrence and outcome, and re-referral rates to gastroenterology, comparing the intervention with usual care. Secondary outcomes include quality of life and global overall symptom score and comparing endoscopy utilization and clinical outcome between primary care and specialty care practices.

### Statistical Analysis

Variables analyzed included age at triage; gender; primary indication defined as IBS, dyspepsia, GERD, abdominal pain or epigastric pain; performance of endoscopy and type (esophagogastroduodenoscopy versus colonoscopy); indication for endoscopy; endoscopic findings; and emergency department visits following initial referral to Central Access and Triage. Medians with interquartile ranges were calculated for wait times from referral to assessment and were compared using the Wilcoxon rank sum test. Similarly, wait times for endoscopy and re-referrals to Central Access and Triage were also compared between pathways. Sensitivity analyses were performed: (1) stratification of our primary analysis by age at enrollment (i.e., <50 or ≥50); (2) a comparison of our primary analyses in the nurse-led, shared medical appointment pathway between patients seen by a primary care physician with an interest in gastroenterology versus a gastroenterologist. The global overall symptom score and SF-12, mental composite summary score and physical component summary score were evaluated at baseline and at six-month follow-up. Matched pairs were analyzed for significance using the Wilcoxon signed rank test.

All statistical analyses were performed using the SAS 9.4 software platform for Windows (SAS Institute, Cary, NC). Statistical tests and comparisons were considered significant at a two-sided P value of <0.05 unless otherwise indicated. The study was approved as a quality improvement initiative by the University of Calgary Research Ethics Board.

## RESULTS

Of the 770 patients evaluated in this study, 53.4% (411 of 770) of patients participated in the nurse-led, shared medical appointment and the remaining 46.6% (359 of 770) of patients in usual care (Table 1). The most common indications for referral in both pathways were GERD and dyspepsia (Table 1). All 411 participants in the intervention pathway received an appointment, with a median wait time of 12.6 weeks. In the usual care pathway, 70.5% of patients were seen by a gastroenterologist as of December 31, 2016, with a significantly longer median wait time, 137.1 weeks (P < 0.0001) (Table 2).

**Table 1.** Cohort demographics

	Nurse-Led Appointment		Usual Care		*P*-value
Number of Patients (N)	411		359		
Female	248 (60.3%)		211 (58.8%)		0.6586
Male	163 (39.7%)		148 (41.2%)		
Median Age (IQR)	44.8 (34.1–55.8)		44.6 (34.6–56.1)		0.7138
Female	45.6 (34.5–57.5)		46.4 (36.8–59.7)		
Male	43.7 (32.7–55.2)		42.3 (32.2–51.5)		
Top 3 Indications for Referral	GERD	198 (48.2%)	GERD	193 (53.8%)	
	Dyspepsia	131 (31.9%)	Dyspepsia	123 (34.3%)	
	IBS	17 (4.1%)	Abdominal Pain	9 (2.5%)	

**Table 2.** Wait times, endoscopic examination, and clinical outcome

	Nurse-Led Appointment		Usual Care		*P*-value
Number of patients (%)	411 (61.9%)		253 (38.1%)		
Median Wait Time to Consult (weeks) (IQR)	12.57 (8.29–21.57)		137.14 (47.57–207.71)		<0.0001
Endoscopy Complete					<0.0001
Yes	209 (50.9%)		193 (76.3%)		
No	202 (49.1%)		60 (23.7%)		
Type of Endoscopic Exam	N = 298		N = 307		
Colonoscopy	100 (33.6%)		90 (29.3%)		
Esophogastroduodenoscopy	184 (61.7%)		203 (66.1%)		
Other*	14 (4.7%)		14 (4.6%)		
Median Wait Time to Endoscopy (weeks) (IQR)	36.86 (23.29–64.14)		65.00 (46.14–131.64)		<0.0001
Top 5 Indications for Endoscopy	**Abdo Pain**	87 (29.2%)	**Dyspepsia**	50 (16.3%)	
	**GERD**	34 (11.4%)	**Abdo Pain**	47 (15.3%)	
	**Dyspepsia**	27 (9.1%)	**Heartburn**	45 (14.7%)	
	**CC Screening**	25 (8.4%)	**CC Screening**	26 (8.5%)	
	**Diarrhea**	20 (6.7%)	**Dysphagia**	25 (8.1%)	
Top 5 Endoscopic Findings	**Normal**	128 (43.0%)	**Normal**	121 (39.4%)	
	**Polyps/Benign**		**Polyps/ Benign**		
	**Neoplasia**	52 (17.4%)	**Neoplasia**	52(16.9%)	
	**Hemorrhoids**	28 (9.4%)	**Esophagitis**	25 (8.1%)	
	**Diverticulosis**	25 (8.4%)	**Diverticulosis**	20 (6.5%)	
	**Gastritis**	22 (7.4%)	**Hiatus Hernia**	18 (5.9%)	
Significant Outcomes^†^	15 (3.6%)		21 (5.8%)		0.1492
Cancer/High Grade Dysplasia	1 (0.2%)		1 (0.3%)		
IBD/Microscopic Colitis	5 (1.2%)		3 (0.8%)		
Esophageal Disease^‡^	10 (2.4%)		11 (3.0%)		
Celiac	0		5 (1.4%)		
Achalasia	1 (0.2%)		1 (0.3%)		
Emergency Department Visits Following Referral to GI Central Triage^†^	25 (6.1%)		43 (12.0%)		0.004
Re-referral to GI Central Triage^†^	19 (4.6%)		56 (15.6%)		<0.0001

* “Other” includes sigmoidoscopy, thin scope endoscopy, and endoscopic ultrasound

^†^ Number of unique patients

^‡^ “Esophageal Disease” includes Barrett**’**s esophagus, Grade C or D esophagitis, eosinophilic esophagitis, and esophageal strictures

Endoscopy was more commonly performed in usual care (76.3%) compared with the nurse-led, shared medical appointment pathway (50.9%) (P < 0.0001) (Table 2). In patients below age 50 and 50 years or older, the usual care pathway had significantly more endoscopic examinations compared with the nurse-led, shared medical appointment pathway (age <50, 41.6% versus 68.6%, P < 0.0001) (age ≥50, 65.2% versus 89.4%, P < 0.0001) (Appendix 5). The most common endoscopic finding in both pathways regardless of age was ‘normal’ (43.0% for the nurse-led, shared medical appointment pathway and 39.4% for usual care), with a low overall rate of significant outcome (3.6% versus 5.8%, P = 0.1492). Similarly, fewer intervention patients (6.1%) compared with usual care visited the Emergency Department (ED) for a related complaint (12.0%) (P = 0.004). A smaller portion of patients in the nurse-led, shared medical appointment pathway (4.6%) was re-referred to GI Central Access and Triage or privately for a related or the same concern compared with usual care (15.6%) (P < 0.0001) (Table 2). Significant differences in ED visits and re-referral for the same clinical issue existed for patients age 50 or above in usual care compared with the nurse-led, shared medical appointment pathway (Appendix 5). There was a higher rate of neoplasia detected in patients at or over 50 years old (Appendix 5).

Baseline and six-month repeat global overall symptom scores were completed by 38.2% of those in the intervention and 16.7% in usual care. Shared appointment participants reported significantly improved scores from baseline (P < 0.0001), whereas no difference was detected in usual care (P = 0.8443) (Table 3). Baseline and repeat SF-12 surveys were completed by 36.5% of patients in the intervention pathway and by 12.8% in usual care. Those in the nurse-led, shared medical appointment pathway had a significant improvement from baseline in the physical component summary score (P = 0.0031) and the mental summary score (P < 0.0001); the usual care pathway did not (P = 0.8200 and P = 0.3517, respectively) (Table 4).

**Table 3.** Global overall symptom scale

	Nurse-Led Appointment	*P*-value	Usual Care	*P*-value
Baseline & Repeat GOS Complete	157 (38.2%)		60 (16.7%)	
Median baseline GOS	4.00		4.00	
Median repeat GOS	3.00		4.00	
Median Change in GOS	1.00	<0.0001	0	0.8443

**Table 4.** Quality of life measures—SF12

	Nurse-Led Appointment	*P*-value	Usual Care	*P*-value
Baseline & Repeat SF12	150 (36.5%)		46 (12.8%)	
Median baseline PCS	51.21		42.88	
Median baseline MCS	46.85		47.80	
Median repeat PCS	53.79		43.60	
Median repeat MCS	50.36		46.77	
Change in SF12				
Median change in PCS	1.00	0.0031	0.44	0.8200
Median change in MCS	3.59	<0.0001	0.13	0.3517

* Physical Component Summary: a measurement of physical health

^†^ Mental Component Summary: a measurement of mental health

After the nurse-led, shared medical appointment pathway, 63.3% were assessed by a gastroenterologist and 36.7% were assessed by a primary care physician (Table 5). There was no difference in patient experience or outcome between patients seen by each provider during the two-year follow-up nor difference in wait time (11.4 weeks versus 14.1 weeks, P = 0.1145), endoscopy rate (52.3% versus 48.3%, P = 0.4384), significant outcome (3.1% versus 4.6%, P = 0.4165), ED visits (6.2% versus 6.0%, P = 0.9369), or rates of re-referral for the same concern (3.5% versus 6.6%, P = 0.1412). When patients were stratified by age, either below 50 years old or 50 years and older, reflecting the national age for initiation of colorectal cancer screening and the recommended age limit for considering gastroscopy in dyspepsia patients, no differences were identified between specialty and primary care practice patterns (i.e., rates of endoscopy, emergency department visits for the same clinical issue or re-referral for the same clinical issue) (Appendix 6).

**Table 5.** Endoscopy rates and outcomes for patients seen by specialty versus primary care in the nurse-led, shared medical appointment cohort

	Gastroenterology		Family Physician		*P*-value
Number of patients (%)	260 (63.3%)		151 (36.7%)		
Median Wait Time to Consult (weeks)	11.43 (7.79–18.50)		14.14 (9.14–25.00)		0.1145
Endoscopy Complete					
Yes	136 (52.3%)		73 (48.3%)		
No	124 (47.7%)		78 (51.7%)		0.4384
Type of Endoscopy	196		102		
Colonoscopy	65 (33.2%)		35 (34.3%)		
Esophagastroduodenoscopy	121 (61.7%)		63 (61.8%)		
Other*	10 (5.1%)		4 (3.9%)		
Median Wait Time to Endoscopy (weeks)	41.21 (23.29–71.79)		33.43 (23.21–45.21)		0.144
Top 5 Indications for Endoscopy	**Abdo Pain**	57 (29.1%)	**Abdo Pain**	30 (29.4%)	
	**Dyspepsia**	22 (11.2%)	**GERD**	17 (16.7%)	
	**CC Screening**	17 (8.7%)	**CC Screening**	8 (7.8%)	
	**GERD**	17 (8.7%)	**Diarrhea**	8 (7.8%)	
	**Diarrhea**	12 (6.1%)	**Dysphagia**	7 (6.9%)	
Top 5 Endoscopic Findings	**Normal**	89 (45.4%)	**Normal**	39 (38.2%)	
	**Polyps/Benign**		**Polyps/Benign**		
	**Neoplasia**	36 (18.4%)	**Neoplasia**	16 (15.7%)	
	**Hemorrhoids**	18 (9.2%)	**Gastritis**	15 (14.7%)	
	**Diverticulosis**	16 (8.2%)	**Hemorrhoids**	9 (8.8%)	
	**Gastritis**	9 (4.6%)	**Diverticulosis**	9 (8.8%)	
Significant Outcomes^†^	8 (3.1%)		7 (4.6%)		0.4165
Cancer/High Grade Dysplasia	0		1 (0.7%)		
IBD/Microscopic Colitis	4 (1.5%)		1 (0.7%)		
Esophageal Disease^‡^	4 (1.5%)		6 (4.0%)		
Celiac	0		0		
Achalasia	0		1 (0.7%)		
Emergency Department Visits Following Referral to GI Central Triage^†^	16 (6.2%)		9 (6.0%)		0.9369
Re-referral to GI Central Triage^†^	9 (3.5%)		10 (6.6%)		0.1412

* “Other” includes sigmoidoscopy, thin scope endoscopy, and endoscopic ultrasound

^†^ Number of unique patients

^‡^ “Esophageal Disease” includes Barrett’s esophagus, Grade C or D esophagitis, eosinophilic esophagitis, and esophageal strictures

## DISCUSSION

This innovative, nurse-led, shared medical appointment pathway for adult gastroenterology patients demonstrates improved workflow with more efficient access to health care and safety, with no worrisome findings identified during two years of follow up. With emphasis on education and self-management (as opposed to the exhaustive investigation of common GI conditions) and a collaborative, multidisciplinary format, patients exhibited symptom improvement and better quality of life. They visited the emergency department less frequently and sought re-referral less often. This model provides evidence for an integrative, specialty-primary care group model to better address the rising demand for gastroenterology services.

Little published data exist that reflect novel approaches including shared medical appointments for gastroenterology, aimed to better address lengthy wait times (18). Overall access to endoscopic investigation is limited in Canada; therefore, a one-on-one 30-minute consultation followed at a later date by endoscopic investigation may present a significant bottleneck for patients with these common, generally benign conditions. There may also be a tendency towards endoscopic investigation, given various incentives and patient expectations, which is an opposing force to current guidelines (19). For example, Choosing Wisely Canada® outlines the most appropriate test and encourages limitation of unnecessary, invasive, costly and potentially harmful investigations that do not lead to better outcomes (20). In this group appointment model, the emphasis is on self-management and empowerment within a positive diagnostic paradigm, thus limiting the need for unnecessary investigations (21).

Shared medical appointments have been credited with the capacity to transform and improve health care service delivery (22). The value add from a group interaction is innumerable, including peer-to-peer support, exchange of additional information beyond the health care team, a sense of solidarity that one is not “alone” in their struggle, just to site a few examples (23). There is also an immense need to shift the focus of care from acute, specialist and hospital-based provision, as much gastroenterology care is currently structured. Here, the specialist was a support within the primary care medical home and part of a team enabling longitudinal, chronic disease care.

## LIMITATIONS

There are limitations to this study. Nurse-led, shared medical appointments were prioritized, which may have significantly skewed the wait times favorably. The difference in wait times is greater than 100 weeks, highlighting the inability for the current system to accommodate requests for consultation. Assignment to the intervention was also nonrandomized, so potential demographic variation may exist between various Primary Care Networks; however, any potential differences are likely minor given existing diversity in the North of Calgary, the site of intervention and the remaining Calgary Zone. The absence of significant clustering in the data is supported by a sensitivity analysis of median wait times stratified by the Primary Care Networks in the control cohort, where differences in the reported outcome were identified (Appendix 7). The Alberta physician funding model and the structure and funding of the Primary Care Networks, including payments for the paraprofessional staff, may be unique to Alberta, limiting the generalizability of the study. Regardless, as this is an observational study with univariate analysis, selection bias and confounding are possible, and a clustered randomized controlled trial would be required to definitively address this. Outcome follow-up was only two years, and longer-term outcomes are important to ensure durability of the safety signal and to ensure absence of significant missed diagnoses, given a more conservative investigational strategy compared with usual care. However, in reported populations with functional disorders, long-term studies reflect similar low rates of disease when patients are appropriately selected (24). Finally, the response rates for patient surveys were low overall but higher in the intervention group (administered by phone) compared with surveys mailed out in the control group. This may contribute to potential response bias and limitation associated with potential interaction in the measurement of dyspepsia and quality of life.

## CONCLUSION

Nurse-led, shared medical appointments conducted for common, nonurgent gastrointestinal disorders such as GERD, dyspepsia and IBS are safe and effective, improve workflow, access and optimize resource utilization, and align practices with existing guidelines that promote a positive diagnostic strategy. These appointments can be highly effective in the management of a number of chronic conditions where health education is central to management; yet few, if any, reports have been published demonstrating the efficacy in common adult GI disorders. Future studies are important to evaluate physician uptake, cost effectiveness and application in those areas where specialty care is limited, including rural sites.

## Supplementary Material

gwy061_suppl_Supplementary_Appendix_1

gwy061_suppl_Supplementary_Appendix_2

gwy061_suppl_Supplementary_Appendix_3

gwy061_suppl_Supplementary_Appendix_4

gwy061_suppl_Supplementary_Appendix_5

gwy061_suppl_Supplementary_Appendix_6

gwy061_suppl_Supplementary_Appendix_7
