# Supplementary figures and images for: Nurse-Led, Shared Medical Appointments for Common Gastrointestinal Conditions—Improving Outcomes Through Collaboration With Primary Care in the Medical Home: A Prospective Observational Study
Source: J Can Assoc Gastroenterol. 2018 Oct 24;3(2):59–66. doi: 10.1093/jcag/gwy061 (PMC7165260; doi:10.1093/jcag/gwy061)

## Appendix 1: Flow Diagram of Study Design

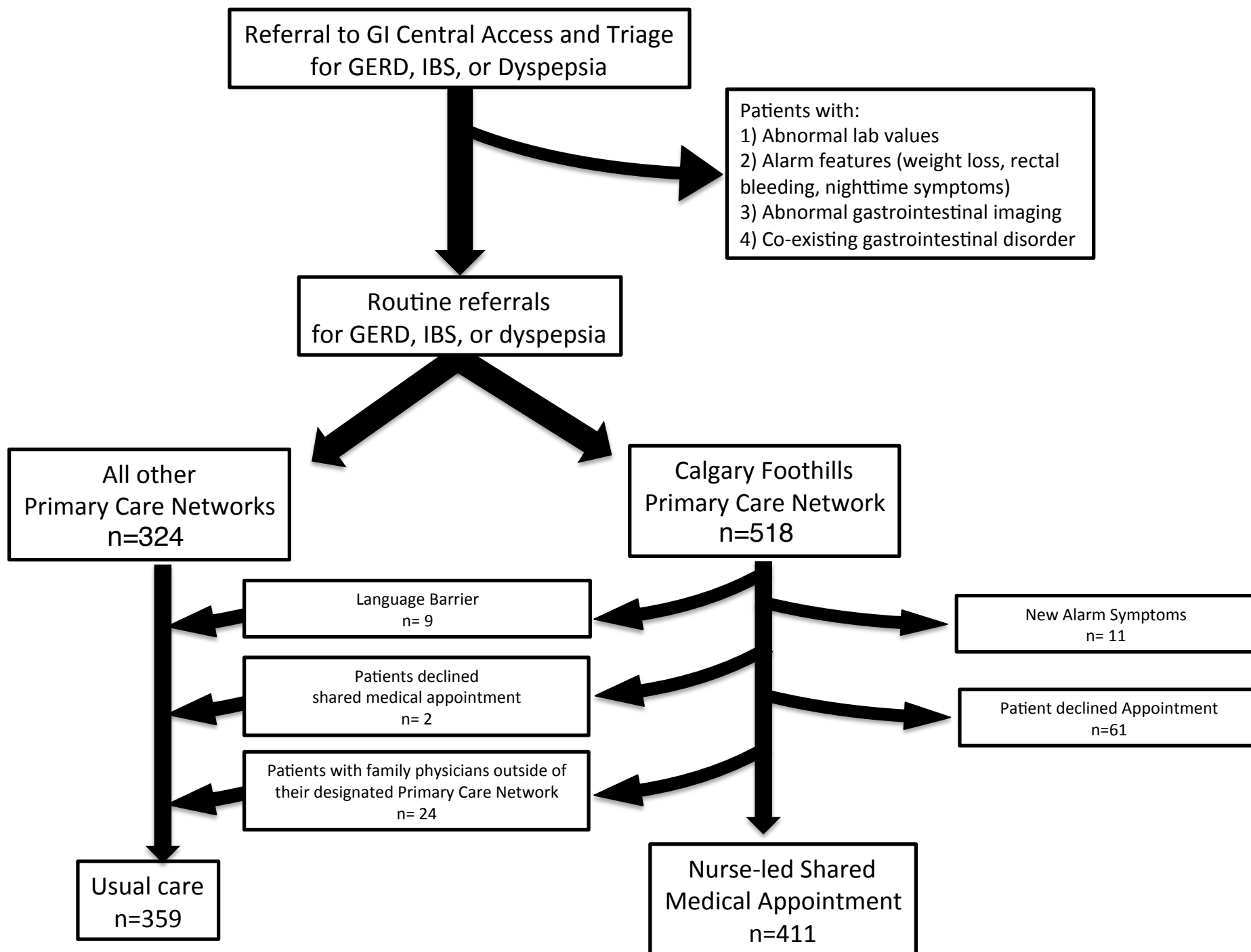

Supplement: gwy061_suppl_Supplementary_Appendix_1 [file gwy061_suppl_supplementary_appendix_1.pdf]
