# Supplementary material for: Nurse-Led, Shared Medical Appointments for Common Gastrointestinal Conditions—Improving Outcomes Through Collaboration With Primary Care in the Medical Home: A Prospective Observational Study
Source: J Can Assoc Gastroenterol. 2018 Oct 24;3(2):59–66. doi: 10.1093/jcag/gwy061 (PMC7165260; doi:10.1093/jcag/gwy061)
Supplement: gwy061_suppl_Supplementary_Appendix_2 [file gwy061_suppl_supplementary_appendix_2.pdf]

Appendix 2: Global Overall Score for Dyspepsia

Rate overall grade overall dyspepsia severity (defined as upper gastrointestinal/ abdominal symptoms located centrally between the breastbone and belly button) over the ***last 28 days*** using the following 7 point scale. Please circle your response.

1. No problem
2. Minimal problem (can be easily ignored without effort)
3. Mild problem (can be ignored with effort)
4. Moderate problem (cannot be ignored but does not influence my daily activities)
5. Moderately severe problem (cannot be ignored and occasionally limits my daily activities)
6. Severe problem (cannot be ignored and often limits my concentration on daily activities)
7. Very severe problem (cannot be ignored and markedly limits my daily activities and often requires rest)

a) epigastric pain (pain in the middle or center of the upper abdomen just below the breastbone),

1. No problem
2. Minimal problem (can be easily ignored without effort)
3. Mild problem (can be ignored with effort)
4. Moderate problem (cannot be ignored but does not influence my daily activities)
5. Moderately severe problem (cannot be ignored and occasionally limits my daily activities)
6. Severe problem (cannot be ignored and often limits my concentration on daily activities)
7. Very severe problem (cannot be ignored and markedly limits my daily activities and often requires rest)

b) epigastric discomfort (same as above; epigastric pain and discomfort are both assessed as some patients perceive these differently)

1. No problem
2. Minimal problem (can be easily ignored without effort)
3. Mild problem (can be ignored with effort)
4. Moderate problem (cannot be ignored but does not influence my daily activities)
5. Moderately severe problem (cannot be ignored and occasionally limits my daily activities)
6. Severe problem (cannot be ignored and often limits my concentration on daily activities)
7. Very severe problem (cannot be ignored and markedly limits my daily activities and often requires rest)

c) heartburn (a burning sensation felt under the lower part of the centre of the chest which rises towards or into the neck)

1. No problem
2. Minimal problem (can be easily ignored without effort)
3. Mild problem (can be ignored with effort)
4. Moderate problem (cannot be ignored but does not influence my daily activities)
5. Moderately severe problem (cannot be ignored and occasionally limits my daily activities)
6. Severe problem (cannot be ignored and often limits my concentration on daily activities)
7. Very severe problem (cannot be ignored and markedly limits my daily activities and often requires rest)

d) acid regurgitation (a backward flow of sour or bitter fluid from the stomach into the food pipe)

1. No problem
2. Minimal problem (can be easily ignored without effort)
3. Mild problem (can be ignored with effort)
4. Moderate problem (cannot be ignored but does not influence my daily activities)
5. Moderately severe problem (cannot be ignored and occasionally limits my daily activities)
6. Severe problem (cannot be ignored and often limits my concentration on daily activities)
7. Very severe problem (cannot be ignored and markedly limits my daily activities and often requires rest)

e) upper abdominal bloating

1. No problem
2. Minimal problem (can be easily ignored without effort)
3. Mild problem (can be ignored with effort)
4. Moderate problem (cannot be ignored but does not influence my daily activities)
5. Moderately severe problem (cannot be ignored and occasionally limits my daily activities)
6. Severe problem (cannot be ignored and often limits my concentration on daily activities)
7. Very severe problem (cannot be ignored and markedly limits my daily activities and often requires rest)

f) excessive belching

1. No problem
2. Minimal problem (can be easily ignored without effort)
3. Mild problem (can be ignored with effort)
4. Moderate problem (cannot be ignored but does not influence my daily activities)
5. Moderately severe problem (cannot be ignored and occasionally limits my daily activities)
6. Severe problem (cannot be ignored and often limits my concentration on daily activities)
7. Very severe problem (cannot be ignored and markedly limits my daily activities and often requires rest)

g) nausea
1. No problem
2. Minimal problem (can be easily ignored without effort)
3. Mild problem (can be ignored with effort)
4. Moderate problem (cannot be ignored but does not influence my daily activities)
5. Moderately severe problem (cannot be ignored and occasionally limits my daily activities)
6. Severe problem (cannot be ignored and often limits my concentration on daily activities)
7. Very severe problem (cannot be ignored and markedly limits my daily activities and often requires rest)

h) early satiety(a feeling the stomach is full soon after starting to eat)
1. No problem
2. Minimal problem (can be easily ignored without effort)
3. Mild problem (can be ignored with effort)
4. Moderate problem (cannot be ignored but does not influence my daily activities)
5. Moderately severe problem (cannot be ignored and occasionally limits my daily activities)
6. Severe problem (cannot be ignored and often limits my concentration on daily activities)
7. Very severe problem (cannot be ignored and markedly limits my daily activities and often requires rest)

i) postprandial fullness (an unpleasant sensation of persistent fullness in the stomach after a meal) and other epigastric symptoms.
1. No problem
2. Minimal problem (can be easily ignored without effort)
3. Mild problem (can be ignored with effort)
4. Moderate problem (cannot be ignored but does not influence my daily activities)
5. Moderately severe problem (cannot be ignored and occasionally limits my daily activities)
6. Severe problem (cannot be ignored and often limits my concentration on daily activities)
7. Very severe problem (cannot be ignored and markedly limits my daily activities and often requires rest)
