# Supplementary material for: Nurse-Led, Shared Medical Appointments for Common Gastrointestinal Conditions—Improving Outcomes Through Collaboration With Primary Care in the Medical Home: A Prospective Observational Study
Source: J Can Assoc Gastroenterol. 2018 Oct 24;3(2):59–66. doi: 10.1093/jcag/gwy061 (PMC7165260; doi:10.1093/jcag/gwy061)
Supplement: gwy061_suppl_Supplementary_Appendix_3 [file gwy061_suppl_supplementary_appendix_3.pdf]

Appendix 3: Short Form 12 Health Survey (SF-12)

SF-12v2

QUALITYMETRIC
HEALTH OUTCOMES SOLUTIONS

SF-12v2™ Health Survey Standard Version

This survey asks for your views about your health. This information will help you keep track of how you feel and how well you are able to do your usual activities. *Thank you for completing this survey!*

For each of the following questions, please click the circle that best describes your answer.

---

1) In general, would you say your health is:

Excellent	Very good	Good	Fair	Poor
○	○	○	○	○

---

2) The following questions are about activities you might do during a typical day. Does your health now limit you in these activities? If so, how much?

	Yes, limited a lot	Yes, limited a little	No, not limited at all
a. Moderate activities, such as moving a table, pushing a vacuum cleaner, bowling, or playing golf	○	○	○
b. Climbing several flights of stairs	○	○	○

---

3) During the past 4 weeks, how much of the time have you had any of the following problems with your work or other regular daily activities as a result of your physical health?

	All of the time	Most of the time	Some of the time	A little of the time	None of the time
a. Accomplished less than you would like	○	○	○	○	○
b. Were limited in the kind of work or other activities	○	○	○	○	○

SF-12v2™ Health Survey Standard Version

4) During the past 4 weeks, how much of the time have you had any of the following problems with your work or other regular daily activities as a result of any emotional problems (such as feeling depressed or anxious)?

	All of the time	Most of the time	Some of the time	A little of the time	None of the time
a. Accomplished less than you would like	○	○	○	○	○
b. Did work or activities less carefully than usual	○	○	○	○	○

5) During the past 4 weeks, how much did pain interfere with your normal work (including both work outside the home and housework)?

Not at all	A little bit	Moderately	Quite a bit	Extremely
○	○	○	○	○

6) These questions are about how you feel and how things have been with you during the past 4 weeks. For each question, please give the one answer that comes closest to the way you have been feeling. How much of the time during the past 4 weeks...

	All of the time	Most of the time	Some of the time	A little of the time	None of the time
a. Have you felt calm and peaceful?	○	○	○	○	○
b. Did you have a lot of energy?	○	○	○	○	○
c. Have you felt downhearted and depressed?	○	○	○	○	○

7) During the past 4 weeks, how much of the time has your physical health or emotional problems interfered with your social activities (like visiting friends, relatives, etc.)?

All of the time	Most of the time	Some of the time	A little of the time	None of the time
○	○	○	○	○
