# Supplementary material for: Nurse-Led, Shared Medical Appointments for Common Gastrointestinal Conditions—Improving Outcomes Through Collaboration With Primary Care in the Medical Home: A Prospective Observational Study
Source: J Can Assoc Gastroenterol. 2018 Oct 24;3(2):59–66. doi: 10.1093/jcag/gwy061 (PMC7165260; doi:10.1093/jcag/gwy061)
Supplement: gwy061_suppl_Supplementary_Appendix_4 [file gwy061_suppl_supplementary_appendix_4.docx]

**Appendix 4: Nurse-led Shared Medical Appointment Outline and Appointment Guide**

Included patients were invited to attend a 1-hour nurse-led shared medical appointment followed by an individual appointment with a physician (gastroenterologist or primary care, duration approximately 15 minutes, including a review to ensure absence of concerning/ red flag symptoms and a physical examination), in lieu of the traditional one-on-one consultation with a gastroenterologist alone. The shared medical appointment was delivered as a single session, including a minimum of 3 and maximum of 8 patient participants. The appointments were conducted in a primary care facility within the Calgary Foothills Primary Care Network and patients were informed the total appointment time approximated to be 1.5-2hrs. All of the staff was employed by the Primary Care Network, with the exception of the gastroenterology nurse and gastroenterologist; both of the gastroenterologists were supported by an academic university-based salary.

The shared medical appointment was facilitated by a gastroenterology-expert nurse and delivered in collaboration with a dietitian, a behavioral health consultant, and a pharmacist.

When patients arrived, they were checked in with height, weight and body mass index (BMI) recorded. The gastroenterology nurse introduced the class and the staff involved in the session. A confidentiality agreement and informed consent form were explained and signed by each participant. The expert nurse followed a shared medical appointment guide, outlining a standard approach that will be summarized here:

1. Introductions and collection of participant topics of interest
2. Didactic epidemiology and pathophysiology
3. Diagnosis and investigation
4. Management
5. Multidisciplinary participation
6. Sharing of resources

Firstly, the nurse conducted a discussion to solicit topics important to the participants, to ensure patient questions/ interests were identified and thus addressed during the session. The nurse then provided a basic overview of the epidemiology and pathophysiology of the three conditions (IBS, dyspepsia and GERD), generated and summarized by the supervising physician (KN).The content was reviewed with nurse to ensure comfort and ease with presentation. Patient’s participation was facilitated throughout. For example, each individuals is asked to name one symptom they experience and at least one approach attempted to reduce symptoms with reports of any associated success to share learnings and foster solidarity. The nurse then provided an overview of additional common symptoms as well as a positive approach to diagnosis based on stereotypic and chronic symptoms, with additional discussion regarding potential indications for further investigations, risks and potential benefits. For example, the role of esophagogastroscopy for the diagnosis and management of GERD. Standard approaches to management were discussed including medical and lifestyle modifications. All facilitated group discussion employed standard adult education principles, with emphasis placed on self-management and empowerment.

The dietitian then provided an overview of common dietary triggers, with instruction of the importance and utility of collecting participant food records to document intake patterns and identify food triggers. Suggested diets were outlined, including FODMAPs = Fermentable Oligosaccharides Disaccharides Monosaccharides and Polyols.

The pharmacist provided an overview of available medications, mechanism of action and optimized use for each condition where appropriate.

The behavioral health consultant then outlined the importance of personal factors, such as stress and mental health in the pathophysiology of these conditions, as well provision of behavior change strategies.

All staff answered questions throughout the appointment as they arose.

At the conclusion of the shared medical appointment, patients were seen by a gastroenterologist or a primary care physician for a one-on-one evaluation. As patients waited for their physician appointment, the multidisciplinary staff could focus on individual questions or concerns.

Additional reference information was provided and included:

- Canadian Digestive Health Foundation – website references for IBS, dyspepsia and GERD
- Canada Food Guide
- Bad Gut.org – the role of stress in chronic functional abdominal conditions
- Food sources of soluble fibre
- Food symptom journal
- *Alberta Health Services materials:* ‘When you have Functional Dyspepsia’; ‘When you have acid reflux (GERD)’; ‘When you have dyspepsia (Indigestion)’; ‘Irritable Bowel Syndrome’; ‘Fibre Facts’
