# Supplementary material for: Nurse-Led, Shared Medical Appointments for Common Gastrointestinal Conditions—Improving Outcomes Through Collaboration With Primary Care in the Medical Home: A Prospective Observational Study
Source: J Can Assoc Gastroenterol. 2018 Oct 24;3(2):59–66. doi: 10.1093/jcag/gwy061 (PMC7165260; doi:10.1093/jcag/gwy061)
Supplement: gwy061_suppl_Supplementary_Appendix_5 [file gwy061_suppl_supplementary_appendix_5.docx]

**Appendix 5: Age Stratified Endoscopic Rates, Clinical Outcomes, Re-referral Rates, and Emergency Department Visits**

|  | Nurse-led Appointment  (< 50 years old) | | Usual Care  (< 50 years old) | | Nurse-led Appointment  (≥ 50 years old) | | Usual Care  (≥ 50 years old) | |
| --- | --- | --- | --- | --- | --- | --- | --- | --- |
| Number of patients (%) | 250 (61%) | | 159 (63%) | | 161 (39%) | | 94 (37%) | |
| Median Wait Time to Consult (weeks) (IQR) | 13.50 (8.43-22.57) | | 58.43^+^ (44.29-146.14) | | 11.14 (7.57-19.43) | | 86.93^++^ (43.29-137.71) | |
| Endoscopy Complete  Yes  No | 104 (41.6%)  146 (58.4%) | | 109 (68.6%)^+^  50 (31.4%) | | 105 (65.2%)  56 (34.8%) | | 84 (89.4%)^++^  10 (10.6%) | |
| Type of Endoscopy  Colonoscopy  Esophogastroduodenoscopy  Other^+++^ | 136  40 (29.4%)  86 (63.2%)  10 (7.4%) | | 160  32 (20.0%)  119 (74.4%)  9 (5.6%) | | 162  60 (37.0%)  98 (60.4%)  4 (2.5%) | | 147  58 (39.5%)  84 (57.1%)  5 (3.4%) | |
| Median Wait Time to Endoscopy (weeks) (IQR) | 36.57 (24.93-57.86) | | 57.57^+^ (46.43-125.93) | | 36.86 (19.43-71.50) | | 95.29^++^ (45.86-136.07) | |
| Top 5 Indications for Endoscopy | **Abdo Pain**  **GERD**  **Dyspepsia**  **Diarrhea**  **Anemia** | 46 (33.8%)  18 (13.2%)  13 (9.6%)  11 (8.1%)  7 (5.1%) | **Dyspepsia**  **Abdo Pain**  **Heartburn**  **GERD**  **Dysphagia** | 31 (19.4%)  29 (18.1%)  28 (17.5%)  15 (9.4%)  14 (8.8%) | **Abdo Pain**  **CC Screening**  **GERD**  **Dysphagia**  **Dyspepsia** | 41 (25.3%)  18 (11.1%)  16 (9.9%)  15 (9.3%)  14 (8.6%) | **CC Screening**  **Dyspepsia**  **Abdo Pain**  **Heartburn**  **Dysphagia** | 21 (14.3%)  19 (12.9%)  18 (12.2%)  17 (11.6%)  11 (7.5%) |
| Top 5 Endoscopic Findings | **Normal**  **Polyps/Benign Neoplasia**  **Hemorrhoids**  **Gastritis**  **Diverticulosis** | 73 (53.7%)  16 (11.8%)  11 (8.1%)  7 (5.1%)  6 (4.4%) | **Normal**  **Esophagitis**  **Hemorrhoids**  **Gastritis**  **Polyps/Benign Neoplasia** | 78 (48.8%)  16 (10.0%)  10 (6.3%)  10 (6.3%)  9 (5.6%) | **Normal**  **Polyps/Benign Neoplasia**  **Diverticulosis**  **Hemorrhoids**  **Gastritis** | 55 (34.0%)  36 (22.2%)  19 (11.7%)  16 (9.9%)  15 (9.3%) | **Normal**  **Polyps/Benign Neoplasia**  **Diverticulosis**  **Hiatus Hernia**  **Esophagitis** | 43 (29.3%)  43 (29.3%)  14 (9.5%)  12 (8.2%)  9 (6.1%) |
| Significant Outcomes*  Cancer/High Grade Dysplasia  IBD/Microscopic Colitis  Esophageal Disease**  Celiac  Achalasia | 7 (2.8%)  0  3 (1.2%)  4 (1.6%)  0  0 | | 15 (6.7%)^+^  0  1 (0.4%)  8 (3.6%)  5 (2.2%)  1 (0.4%) | | 8 (5.0%)  1 (0.6%)  2 (1.2%)  6 (3.7%)  0  1 (0.6%) | | 6 (4.4%)  1 (0.7%)  2 (1.5%)  3 (2.2%)  0  0 | |
| Emergency Department Visits Following Referral to GI Central Triage* | 20 (8.0%) | | 26 (11.7%) | | 5 (3.1%) | | 17 (12.5%)^++^ | |
| Re-referral to GI Central Triage* | 10 (4.0%) | | 31 (13.9%)^+^ | | 9 (5.6%) | | 25 (18.4%)^++^ | |
| + Significant difference exists when comparing the cohorts of patients < 50 years old.  ++ Significant difference exists when comparing the cohorts of patients ≥ 50 years old.  (Only those comparisons with statistically significant differences are identified.)  +++ “Other” includes sigmoidoscopy, thin scope endoscopy, and endoscopic ultrasound.  * Number of unique patients.  ** “Esophageal Disease” includes Barrett’s esophagus, Grade C or D esophagitis, eosinophilic esophagitis, and esophageal strictures. | | | | | | | | |
