# Supplementary material for: Nurse-Led, Shared Medical Appointments for Common Gastrointestinal Conditions—Improving Outcomes Through Collaboration With Primary Care in the Medical Home: A Prospective Observational Study
Source: J Can Assoc Gastroenterol. 2018 Oct 24;3(2):59–66. doi: 10.1093/jcag/gwy061 (PMC7165260; doi:10.1093/jcag/gwy061)
Supplement: gwy061_suppl_Supplementary_Appendix_6 [file gwy061_suppl_supplementary_appendix_6.docx]

**Appendix 6: Endoscopy Rates and Outcomes for Patients Seen by Specialty Care vs. Primary Care, Stratified by Age in the Nurse-Led Shared Medical Appointment Cohort**

|  | Gastroenterology  (< 50 years old) | | Family Physician  (< 50 years old) | | Gastroenterology  (≥ 50 years old) | | Family Physician  (≥ 50 years old) | |
| --- | --- | --- | --- | --- | --- | --- | --- | --- |
| Number of patients (%) | 161 (39.2%) | | 89 (21.7%) | | 99 (24.1%) | | 62 (15.1%) | |
| Median Wait Time to Consult (weeks) | 12.14 (8.00-21.21) | | 15.00^+^ (10.00-27.29) | | 11.14 (7.29-17.29) | | 11.29 (8.57-21.57) | |
| Endoscopy Complete  Yes  No | 70 (43.5%)  91 (56.5%) | | 34 (38.2%)  55 (61.8%) | | 66 (66.7%)  33 (33.3%) | | 39 (62.9%)  23 (37.1%) | |
| Type of Endoscopy  Colonoscopy  Esophogastroduodenoscopy  Other^++^ | 91  25 (27.5%)  57 (62.6%)  9 (9.9%) | | 45  15 (33.3%)  29 (64.4%)  1 (2.2%) | | 105  40 (38.1%)  64 (61.0%)  1 (1.0%) | | 57  20 (35.1%)  34 (59.6%)  3 (5.3%) | |
| Median Wait Time to Endoscopy (weeks) | 37.86 (24.00-61.86) | | 34.00 (27.86-50.29) | | 44.86 (20.14-81.86) | | 33.43 (19.00-43.43) | |
| Top 5 Indications for Endoscopy | **Abdo Pain**  **GERD**  **Dyspepsia**  **CC Screening**  **Bright Red Bleeding** | 35 (38.5%)  10 (11.0%)  10 (11.0%)  5 (5.5%)  5 (5.5%) | **Abdo Pain**  **GERD**  **Diarrhea**  **Dysphagia**  **Iron Deficiency** | 11 (24.4%)  8 (17.8%)  4 (8.9%)  4 (8.9%)  4 (8.9%) | **Abdo Pain**  **CC Screening**  **Dyspepsia**  **Dysphagia**  **GERD** | 22 (21.0%)  12 (11.4%)  12 (11.4%)  11 (10.5%)  7 (6.7%) | **Abdo Pain**  **GERD**  **CC Screening**  **Diarrhea**  **Dysphagia** | 19 (33.3%)  9 (15.8%)  7 (12.3%)  4 (7.0%)  3 (5.3%) |
| Top 5 Endoscopic Findings | **Normal**  **Polyps/Benign Neoplasia**  **Hemorrhoids**  **Esophagitis**  **Diverticulosis** | 53 (58.2%)  11 (12.1%)  5 (5.5%)  4 (4.4%)  3 (3.3%) | **Normal**  **Gastritis**  **Hemorrhoids**  **Polyps/Benign Neoplasia**  **Diverticulosis** | 20 (44.4%)  8 (17.8%)  6 (13.3%)  5 (11.1%)  3 (6.7%) | **Normal**  **Polyps/Benign Neoplasia**  **Hemorrhoids**  **Diverticulosis**  **Gastritis** | 36 (34.3%)  25 (23.8%)  13 (12.4%)  13 (12.4%)  8 (7.6%) | **Normal**  **Polyps/Benign Neoplasia**  **Gastritis**  **Diverticulosis**  **Hemorrhoids** | 19 (33.3%)  11 (19.3%)  7 (12.3%)  6 (10.5%)  3 (5.3%) |
| Significant Outcomes*  Cancer/High Grade Dysplasia  IBD/Microscopic Colitis  Esophageal Disease**  Celiac  Achalasia | 4 (2.5%)  0  3 (1.9%)  1 (0.6%)  0  0 | | 3 (3.4%)  0  0  3 (3.4%)  0  0 | | 4 (4.0%)  0  1 (1.0%)  3 (3.0%)  0  0 | | 4 (6.5%)  1 (1.6%)  1 (1.6%)  3 (4.8%)  0  1 (1.6%) | |
| Emergency Department Visits Following Referral to GI Central Triage* | 13 (8.1%) | | 7 (7.9%) | | 3 (3.0%) | | 2 (3.2%) | |
| Re-referral to GI Central Triage* | 5 (3.1%) | | 5 (5.6%) | | 4 (4.0%) | | 5 (8.1%) | |
| + Significant difference exists when comparing the cohorts of patients < 50 years old.  (Only those comparisons with statistically significant differences are identified.)  ++ “Other” includes sigmoidoscopy, thin scope endoscopy, and endoscopic ultrasound.  * Number of unique patients.  ** “Esophageal Disease” includes Barrett’s esophagus, Grade C or D esophagitis, eosinophilic esophagitis, and esophageal strictures. | | | | | | | | |
