# Supplementary material for: Nurse-Led, Shared Medical Appointments for Common Gastrointestinal Conditions—Improving Outcomes Through Collaboration With Primary Care in the Medical Home: A Prospective Observational Study
Source: J Can Assoc Gastroenterol. 2018 Oct 24;3(2):59–66. doi: 10.1093/jcag/gwy061 (PMC7165260; doi:10.1093/jcag/gwy061)
Supplement: gwy061_suppl_Supplementary_Appendix_7 [file gwy061_suppl_supplementary_appendix_7.docx]

Appendix 7: Sensitivity analysis of median wait time to consult comparing the intervention Primary Care Network to each control Primary Care Network

| **Primary Care Network** | **Median Wait Time to Consult (weeks)** | **p value** |
| --- | --- | --- |
| **Foothills (n=35)** | 146.14 (52.43-214.57) | <0.001 |
| **West Central (n=74)** | 142.21 (50.00-206.71) | <0.001 |
| **Highland (n=24)** | 155.93 (47.00-213.86) | <0.001 |
| **Mosaic (n=90)** | 140.21 (49.00-207.43) | <0.001 |
| **South (n=83)** | 84.86 (43.29-208.00) | <0.001 |
| **Rural (including both Calgary Rural and Bow Valley) (n=53)** | 106.71 (48.57-206.71) | <0.001 |
